# Disruption of endothelial adherens junctions by high glucose is mediated by protein kinase C-β–dependent vascular endothelial cadherin tyrosine phosphorylation

**DOI:** 10.1186/1475-2840-13-105

**Published:** 2014-07-15

**Authors:** Mehran Haidari, Wei Zhang, James T Willerson, Richard AF Dixon

**Affiliations:** 1 Department of Internal Medicine, Division of Cardiology, The University of Texas Medical School at Houston, 77030 Houston, TX, USA; 2 Texas Heart Institute at St. Luke’s Episcopal Hospital, PO Box 20345 C1000, 77030 Houston, TX, USA

**Keywords:** Adherens junction, Endothelium, Wnt pathway, Protein kinase C (PKC), Diabetes

## Abstract

**Background:**

Hyperglycemia has been recognized as a primary factor in endothelial barrier dysfunction and in the development of micro- and macrovascular diseases associated with diabetes, but the underlying biochemical mechanisms remain elusive. Tyrosine phosphorylation of vascular endothelial cadherin (VE-cad) leads to the disruption of endothelial adherens junctions and increases the transendothelial migration (TEM) of leukocytes.

**Methods:**

VE-cad tyrosine phosphorylation, adherens junction integrity and TEM of monocytes in human umbilical vein endothelial cells (HUVECs) treated with high-concentration glucose were evaluated. The role of protein kinase C (PKC) in induction of endothelial cells adherence junction disruption by exposure of HUVECs to high concentration of glucose was explored.

**Results:**

The treatment of HUVEC with high-concentration glucose increased VE-cad tyrosine phosphorylation, whereas mannitol or 3-O-methyl-D-glucose had no effect. In addition, high-concentration glucose increased the dissociation of the VE-cad–β-catenin complex, activation of the Wnt/β-catenin pathway, and the TEM of monocytes. These alterations were accompanied by the activation of endothelial PKC and increased phosphorylation of ERK and myosin light chain (MLC). High-concentration glucose-induced tyrosine phosphorylation of VE-cad was attenuated by: 1- the inhibition of PKC-β by overexpression of dominant-negative PKC-β 2- inhibition of MLC phosphorylation by overexpression of a nonphosphorylatable dominant-negative form of MLC, 3- the inhibition of actin polymerization by cytochalasin D and 4- the treatment of HUVECs with forskolin (an activator of adenylate cyclase).

**Conclusions:**

Our findings show that the high-concentration glucose-induced disruption of endothelial adherens junctions is mediated by tyrosine phosphorylation of VE-cad through PKC-β and MLC phosphorylation.

## Introduction

Atherogenesis is a vascular inflammatory process characterized by the enhanced recruitment of leukocytes to dysfunctional endothelium [1]. An important early event preceding the development of atheromatous lesions is the infiltration of the arterial subendothelial intima by macrophages/monocytes [2]. Interendothelial adherens junctions (AJs) that maintain endothelial barrier function are largely composed of vascular endothelial cadherin (VE-cad), an endothelium-specific member of the cadherin family of adhesion proteins. Through its cytoplasmic domain, VE-cad binds to several proteins including p120, β-catenin, and plakoglobin [3,4] that in turn bind *α*-catenin, which links VE-cad to the actin cytoskeleton[5]. Tyrosine phosphorylation of VE-cad disrupts endothelial AJs by causing the dissociation of catenins from VE-cad and thus facilitates the diapedesis of leukocytes [6,7]. Myosin light chain (MLC) phosphorylation has a critical role in increasing the permeability of endothelial cells [8]. Recently, we showed that MLC phosphorylation mediates the tyrosine phosphorylation of VE-cad induced by the interaction of monocytes with endothelial cells, leading to the enhanced transendothelial migration (TEM) of monocytes [9]. Accelerated atherosclerosis is the leading cause of morbidity and mortality in diabetic patients [10]. Hyperglycemia has been implicated as an underlying cause of cardiovascular, retinal, and renal complications in diabetic patients; however, the mechanisms are not known. Hyperglycemia increases the adhesion of leukocytes to endothelial cells [11] possibly as the result of the stimulatory effect of hyperglycemia on the expression of adhesion molecules on the surface of endothelial cells [12,13]. In addition, high glucose levels increase the *in vitro* TEM of monocyte-like HL-60 cells [14], although this effect has been attributed to an increase in the phosphorylation of platelet-endothelial cell adhesion molecule-1(PECAM-1). It has been demonstrated that high concentration of glucose mediates an increase in permeability of endothelial cells to albumin by activation of protein kinase C [15]. However, the integrity of AJs is a characteristic of endothelial barrier that regulates TEM of monocytes [6,7] and might be independent of permeability of endothelial cells to albumin. The molecular mechanisms underlying the effect of high concentration of glucose on integrity of endothelial AJs and TEM of monocytes has not been fully explored. In our study, we hypothesized that high concentrations of glucose disintegrate endothelial AJs and facilitate the TEM of monocytes through the protein kinase C-mediated tyrosine phosphorylation of VE-cad.

## Materials and methods

### Reagents

Phospho-specific and nonphospho-specific antibodies against Src (pY416), proline-rich tyrosine kinase-2 (Pyk2) (pY402), glycogen synthase kinase 3-β (GSK3β, Ser 9), β-catenin, ERK1/2, MYPT, GAPDH and cyclin D1 were purchased from Abcam. Phospho-specific and nonphospho-specific antibodies against VE-cadherin (Y731) and monoclonal 4G10 anti-phosphotyrosine Ab were purchased from Invitrogen. Monoclonal phospho-antibody against MLC and specific antibody against MLC were purchased from Sigma-Aldrich. Mouse IgG was purchased from Southern Biotech. Cytochalasin D, 3-O-methyl-D-glucose, mannitol, D-glucose, phorbol myristate acetate (PMA), forskolin, and protein kinase A inhibitor H89 were purchased from Calbiochem. Premade recombinant adenoviruses for dominant-negative (DN)-protein kinase C (PKC)-α, null control, and green fluorescent protein (GFP) and ViraDuctin adenovirus transduction reagents were purchased from Cell Biolabs, Inc. Premade recombinant adenoviruses for DN-PKC-β and DN-PKC-δ were purchased from Applied Biological Materials. The efficacy of all recombinant adenoviruses was previously tested [9,16,17]. The luciferase reporters, which contain either wild-type (TOPflash) or mutated (FOPflash) binding sites for the lymphoid enhancer factor-1/T-cell specific transcription factor (Lef-1/TCF) complex were purchased from Millipore. The mutant construct for MLC, in which Thr18 and Ser19 were replaced with alanines (A-A-MLC) so that the protein could not be phosphorylated, was a generous gift from Dr. Andreas Kapus, University of Toronto, Toronto, Canada, [18].

### Cells

Human umbilical vein endothelial cells (HUVECs) and human acute monocytic leukemia (THP-1) cells were purchased from American Type Culture Collection. Human aortic endothelial cells (HAECs) were purchased from Lonza. HUVECs and HAECs were grown in Lonza’s EGM-2-MV medium on collagen-coated (20 μg/ml) tissue culture dishes. HUVECs and HAECs from fewer than 4 generations were used for all experiments. THP-1 cells were maintained in Roswell Park Memorial Institute (RPMI) 1640 medium supplemented with 10% heat-inactivated fetal calf serum (FCS). A single-donor human peripheral blood buffy coat was purchased from the Gulf Coast Regional Blood Center (Houston, TX, USA) and used for isolation of peripheral blood monocytes.

### Western blotting

HUVECs were grown to confluency in 35-mmol/l dishes or 6-well plates. Cells were extracted in radioimmunoprecipitation assay (RIPA) buffer, which contained 0.1% SDS, 1% deoxycholate, 1% NP-40, 10 mmol/l sodium phosphate, 150 mmol/l NaCl, 2 mmol/l EDTA, 50 mmol/l NaF, 5 mmol/l sodium pyrophosphate, 0.1 mmol/l sodium vanadate, 2 mmol/l PMSF, 0.1 mg/ml leupeptin, and 100 KIU/ml aprotinin. Samples were loaded onto an SDS-PAGE gel and run at 150 V for 1 h. The proteins were then transferred onto nitrocellulose paper at 300 mA for 1.5 h, followed by Western blot analysis. Blots were blocked with 5% dry milk in 0.1% Tween 20 in PBS for 1 h at room temperature. The primary antibodies were used at a dilution of 1:500 to 1:1000. All antibodies were added for 1 h at room temperature or overnight at 4°C. After washing, the appropriate secondary antibodies (Pierce Biotechnology, Inc., Rockford, IL, USA) were added at a dilution of 1:10 000 for 1 h at room temperature. After extensive washing, blots were developed with the Super Signal enhanced chemiluminescence kit (Pierce) and visualized on Kodak-AR film.

### Immunoprecipitation

Cells were grown to confluency, washed gently with ice-cold PBS twice, and lysed in 1 ml of RIPA lysis buffer. After 10 min on ice, cell lysates were collected and precleared for 30 min at 4°C with protein A-agarose. After centrifugation (14 000 × *g*, 15 s at 4°C), the supernatants were incubated with primary antibodies (1 μg/mg lysate) overnight at 4°C with continuous mixing. Protein A-agarose (40 μl) was added to the lysate. After 2 h at 4°C, the beads were washed 3 times in lysis buffer, and proteins were eluted by boiling in SDS-sample buffer containing 4% 2-mercaptoethanol (Bio-Rad, Hercules, CA, USA). The samples were analyzed by SDS-PAGE.

### Transduction of adenovirus

The conditions used for the transduction of recombinant adenoviruses were optimized by using adenovirus encoding GFP. All reagents and kits, including transduction reagents, an adenovirus purification kit, and an adenovirus titration kit, were purchased from Cell Biolabs, Inc. After purification, the titration of each recombinant adenovirus was determined by an ELISA titrating kit. HUVECs were seeded into 6-well plates for 24 h until they reached 80% confluence. According to the manufacturers’ protocol, adenovirus was transduced into cells by using ViraDuctin (Cell Biolabs, Inc.). HUVECs were infected with adenoviral vectors with a multiplicity of infection (MOI) of 100 plaque-forming units per cell in the presence of ViraDuctin. After incubation with viral particles for 48 h, the cells were assessed for the expression of the transduced genes.

### Transfection of plasmids

The vector pcDNA3.1/CT-GFP TOPO (Invitrogen) was used to optimize the transfection of plasmids into HUVECs. Plasmids were transfected into cells by using Lipofectamine 2000 (Invitrogen). Empty pcDNA3.1 vector was transfected as controls. Cells were collected 48 h after transfection with plasmids.

### Immunofluorescence studies

Cells were grown in wells of 4-chamber culture collagen-coated slides. Cells were fixed in 4% paraformaldehyde for 15 min at 4°C, washed with PBS, and permeabilized for 5 min with 0.1% Triton-X-100. After blocking with PBS + 2% BSA + 0.1% Tween-20, cells were incubated with primary antibody against β-catenin and goat anti-rabbit IgG for 45 min each. Images were acquired by MicroSuite FIVE software (Olympus Soft Imaging Solutions, Golden, CO, USA) with an Olympus BX61 motorized microscope (Olympus America, Center Valley, PA, USA).

### TEM assay

A kit from Cell Biolabs, Inc. was used for TEM assays according to the manufacturer’s instructions. THP-1 cells (25 × 10^6^ each) were resuspended in 1 ml of complete medium and incubated for 1 h at 37°C in the presence of 50 μg/ml calcein-AM (Molecular Probes, Invitrogen). After the cells were labeled, they were resuspended at a concentration of 1 × 10^6^ cell/ml in DMEM. The lower chamber was filled with assay medium plus 10 ng/ml of monocyte chemotactic protein (MCP)-1. THP-1 cells (1.5 × 10^5^ in 150 μl) were added to the upper compartment of transwell chambers with 6.5-mm diameter and 5-μm pores for 2 h. The cells that spontaneously detached from the undersurface of the filter were removed by the swab and were quantified with an Ultra384 plate reader (Tecan) by using 485 and 535 nm excitation and emission filters, respectively.

### Luciferase reporter assay

Briefly, HUVECs (1.5 × 10^4^) were seeded in a 12-well plate and were transiently transfected with 0.3 μg of TOPflash or FOPflash along with 10 ng of pRL-SV40 (Promega) by using Lipofectamine 2000 (Invitrogen). After 48 h, the cells were treated with glucose (12.5, 25, or 50 mM, 24 h), PMA (50 nM, 8 h), LiCl (10 mM, 8 h). Cells were then lysed, and luciferase activity was determined by using a Dual-Luciferase® Reporter Assay System (Promega). The luciferase activity of each sample was normalized with that of Renilla, used for monitoring transfection efficiency.

### PKC total activity

PKC activity was measured by using a MESACUP protein kinase assay kit (MBL International), which is based on an enzyme-linked immunosorbent assay, according to the manufacturer’s protocol. Briefly, subconfluent HUVECs were washed 3 times with cold phosphate-buffered saline (PBS), scraped with a rubber policeman, and suspended in 1 mL of cold sample preparation buffer (50 mM Tris–HCl [pH 7.5], 5 mM EDTA, 10 mM EGTA, 50 mM 2-mercaptoethanol, 1 mM PMSF, 10 mM benzamidine). The phosphatidylserine (PS) peptide plate was phosphorylated by incubation with 30 μg/mL cell lysate, 3 mM MgCl_2_, 2 mM CaCl_2_, 0.1 mM ATP, 50 μg/mL phosphatidylserine, 0.5 mM EDTA, 1 mM EGTA, 5 mM 2-mercaptoethanol, and 25 mM Tris–HCl (pH 7.0) at 25°C for 10 min. The biotinylated monoclonal antibody 2B9 was used to bind the phospho-PS peptide and was subsequently detected with peroxidase-conjugated streptavidin. A peroxidase substrate was then added to the microwells, and color intensity was measured spectrophotometrically at 492 nm.

### Statistics

Transmigration data were analyzed by analysis of variance (ANOVA), and a 2-sample Student *t* test was used to calculate statistical significance (Excel, Microsoft Corp., Houston, TX, USA). All experiments were repeated at least 3 times. A probability (*P*) value <0.05 was considered significant.

## Results

### Treatment of Endothelial Cells with High-Concentration Glucose Leads to the Disintegration of Endothelial AJs

**Figure 1:**
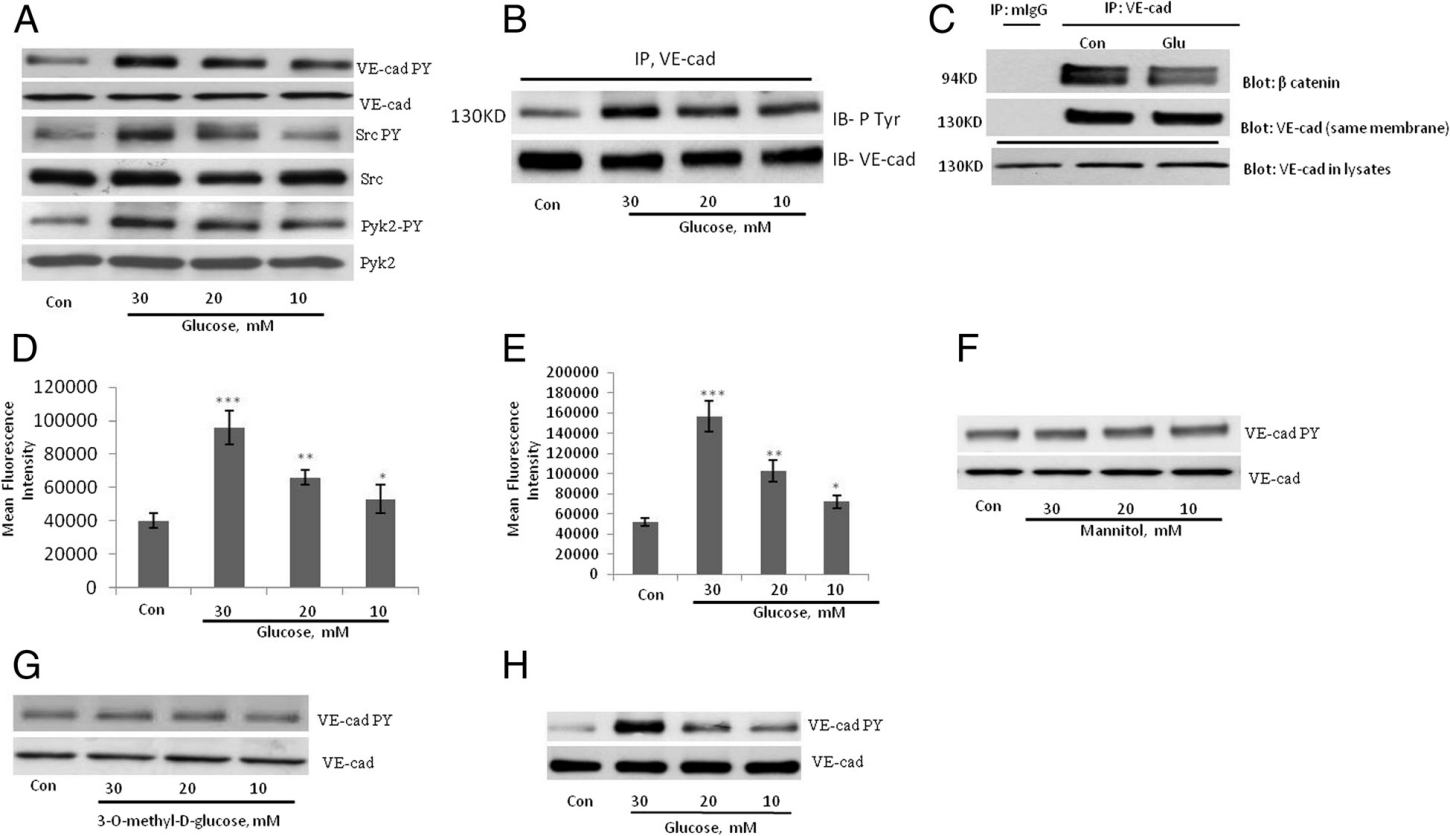
**Treatment of endothelial cells with high-concentration glucose leads to the disintegration of adherens junctions. A**, Tyrosine phosphorylation of VE-cad (PY731), Src (PY416), and Pyk2 (PY402) was induced in human umbilical vein endothelial cells (HUVECs) after treatment with glucose for 24 h using phosphospecific antibodies. **B**, Treatment of HUVECs with high concentration of glucose leads to tyrosin phosphorylation of VE-cadherin, VE-caherin was immunoprecipitated and then blotted with 4G10 anti phospho-tyrosine antibody. **C**, Treatment of HUVECs with 20 mM glucose for 24 h led to the dissociation of β-catenin from VE-cad. VE-cad antibody was used for immunoprecipitation, and β-catenin and VE-cad antibodies were used for the detection of each respective protein. mIgG (mouse non-immune IgG) was used as an immunoprecipitation control. **D**, **E**, Treatment of HUVECs with the indicated concentrations of glucose for 24 h increased the transendothelial migration (TEM) of THP-1 cells and primary human peripheral blood monocytes. TEM of THP-1 cells **(D)** and primary human peripheral blood monocytes **(E)** were evaluated by using a transwell assay. Calcein-labeled THP-1 cells/ primary human peripheral blood monocytes were quantified by using a fluorescence plate reader. Data are expressed as the mean ± SD from triplicate experiments. **F**, **G**, Treatment of HUVECs with the indicated concentrations of mannitol and *3*-*O*-*methyl*-*D glucose* for 24 h did not significantly affect the tyrosine phosphorylation of VE-cad. **H**, Treatment of human aortic endothelial cells with the indicated concentrations of glucose for 24 h led to tyrosine phosphorylation of VE-cad. *P < 0.05, **P < 0.01, ***P < 0.001 vs. control. Each experiment was independently performed 3 to 4 times.

The treatment of HUVECs with high concentrations of D-glucose, otherwise referred to as glucose, for 24 hours increased tyrosine phosphorylation of VE-cad in a dose-dependent manner (Figure 1A). In addition, phosphorylation of the tyrosine kinases Src and Pyk2 that regulate the tyrosine phosphorylation of VE-cad was also increased in HUVECs treated with high concentrations of glucose (Figure 1A). To confirm tyrosine phosphorylation of VE-cadherin after treatment of HUVECs with high glucose VE-cadherin was immunoprecipitated and then tyrosine phosphorylation was tested with monoclonal 4G10 anti-phosphotyrosine Ab. As indicated in Figure 1B tyrosine residues of VE-cadherin was phosphorylated after treatment of HUVECs with high concentration of glucose. When HUVECs were treated with 25 mM glucose, the VE-cad–β-catenin complex was dissociated (Figure 1C). In addition, the TEM of THP-1 cells (a monocytic leukemia cell line) was significantly increased in a dose-dependent manner in HUVECs treated with high concentrations of glucose (Figure 1D). Similar studies with primary human peripheral blood monocytes showed comparable results, indicating that these findings are not specific to THP-1 cells (Figure 1E). In contrast to high-concentration glucose, neither mannitol nor 3-O-methyl-D glucose (a nonmetabolizable glucose analogue) altered VE-cad tyrosine phosphorylation in HUVECs after 24 hours (Figure 1F and G). Similar to HUVECs treated with high concentrations of glucose, human aortic endothelial cells (HAECs) treated with high concentrations of glucose for 24 h also showed a dose-dependent increase in VE-cad tyrosine phosphorylation (Figure 1H). These results suggest that high-concentration glucose disrupts the integrity of endothelial AJs, which is associated by an increase in the tyrosine phosphorylation of VE-cad.

### High-Concentration Glucose–Induced VE-cad Tyrosine Phosphorylation Is Mediated by PKC-β

**Figure 2:**
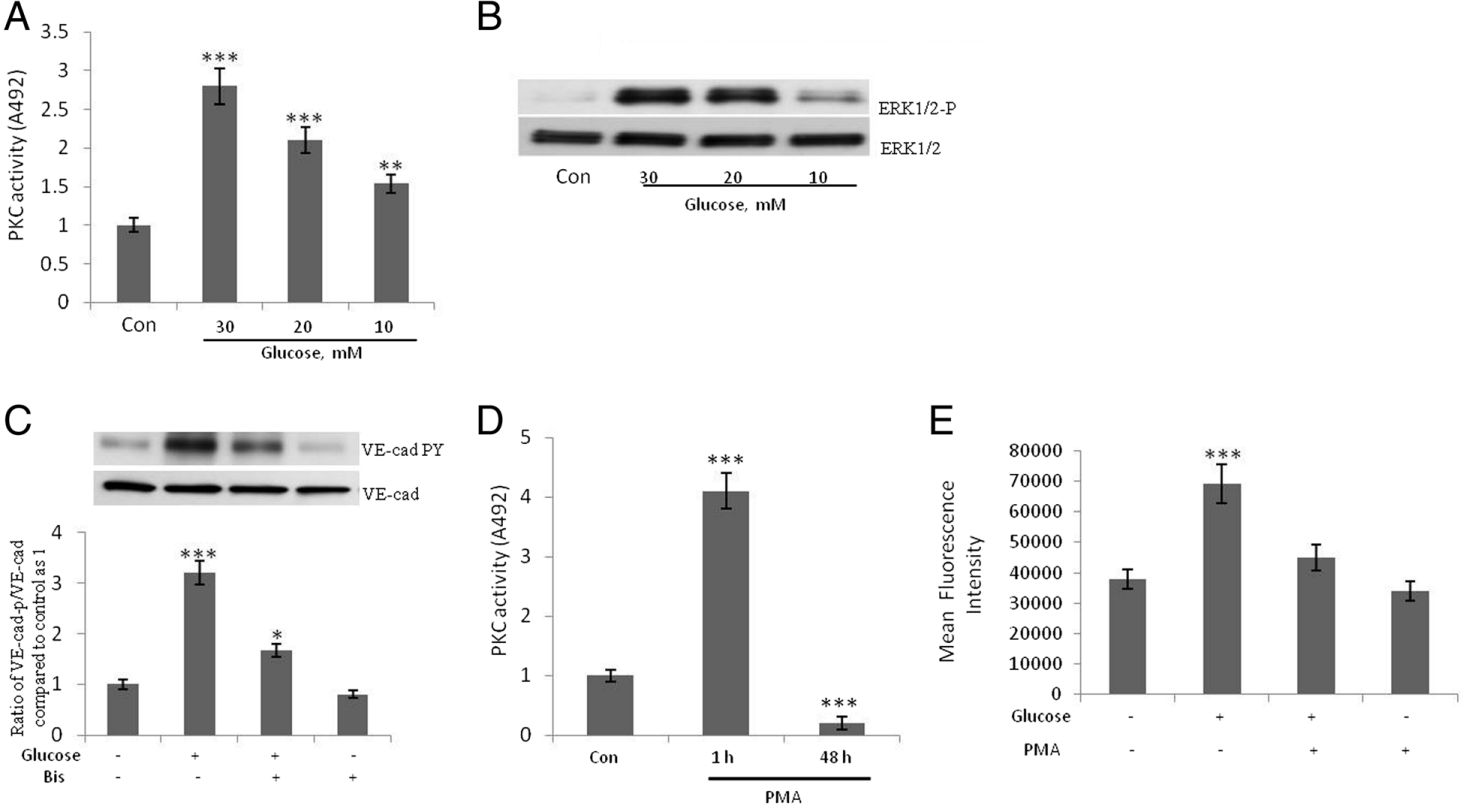
**Protein kinase C (PKC) plays a role in the high-concentration glucose-induced disruption of endothelial adherens junctions. A**, Treatment of human umbilical vein endothelial cells (HUVECs) with glucose for 24 h led to an increase in total PKC activity. Total PKC activity was measured spectrophotometrically at 492 nm by using a colorimetric enzyme-linked immunosorbent assay technique. For each sample shown, three independent experiments were performed. **B**, Treatment of HUVECs with glucose for 24 h increased ERK1/2 phosphorylation. **C**, Treatment of HUVECs with 10 μM PKC inhibitor, bisindolylmaleimide I (Bis), for 2 h attenuated high-concentration glucose-induced tyrosine phosphorylation of VE-cad. HUVECs were first treated with 20 mM of glucose for 24 h. **D**, Treatment of HUVECs for 1 and 48 h increased and inhibited PKC activity, respectively. Total PKC activity was measured spectrophotometrically at 492 nm by using a colorimetric enzyme-linked immunosorbent assay technique. **E**, Treatment of HUVECs with 50 nM of phorbol myristate acetate (PMA) for 48 h attenuated high-concentration glucose-induced transendothelial migration (TEM) of THP-1 cells. HUVECs were washed with the cell culture medium before addition of THP cells. The TEM of THP-1 cells were evaluated by using a transwell assay. Calcein-labeled THP-1 cells were quantified by using a fluorescent plate reader. Data are expressed as the mean ± SD from triplicate experiments. *P < 0.05, **P < 0.01, ***P < 0.001 vs. control. Each experiment was independently performed 3 to 4 times.

**Figure 3:**
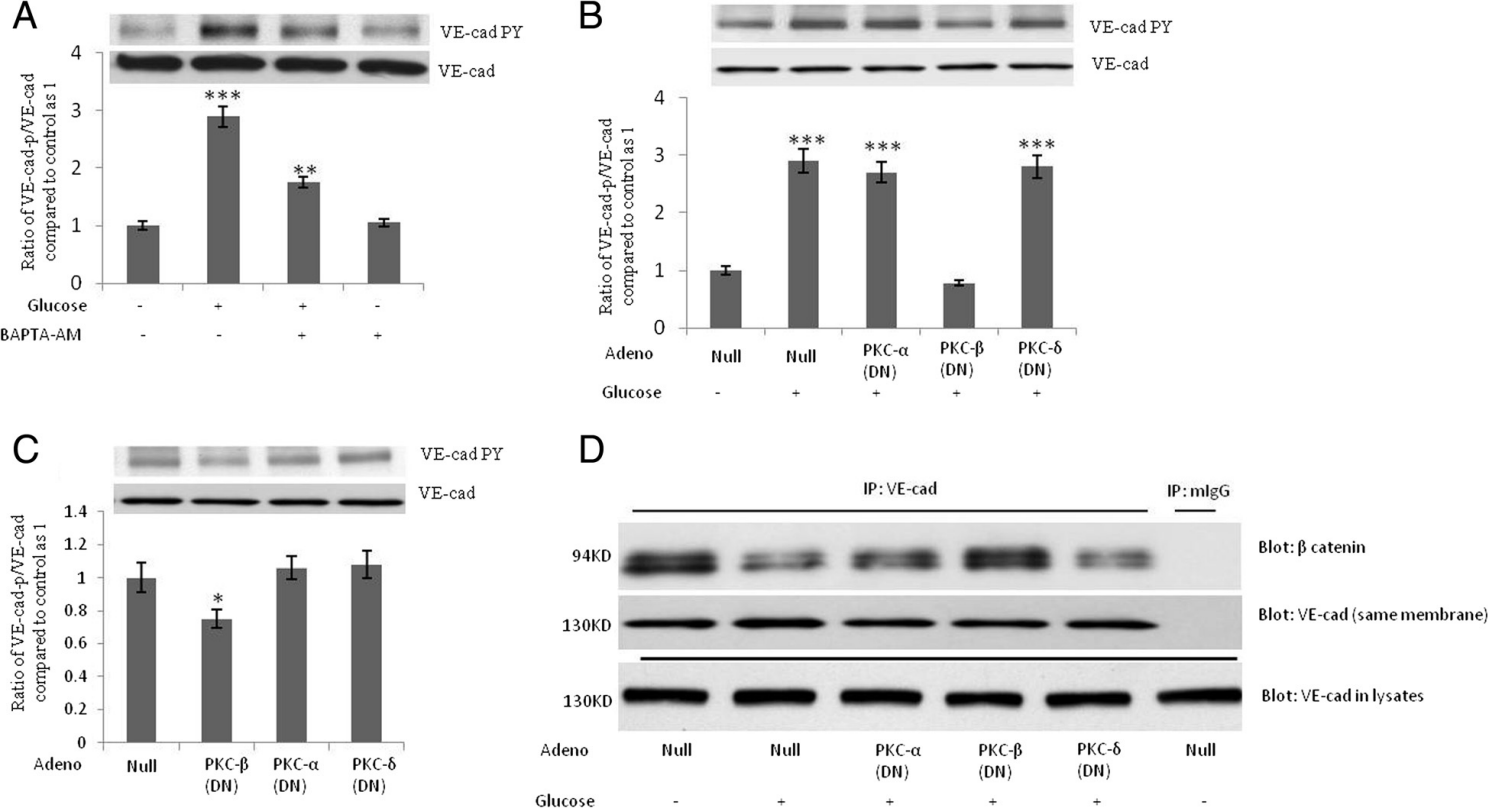
**High-concentration glucose-induced tyrosine phosphorylation of VE-cad is mediated by protein kinase C (PKC)-b. A**, Treatment of human umbilical vein endothelial cells (HUVECs) with 10 μM of cell-permeant intracellular calcium chelator, BAPTA-AM, for 2 h attenuated high-concentration glucose-induced tyrosine phosphorylation of VE-cad. HUVECs were first treated with 20 mM of glucose for 24 h. **B**, Overexpression of recombinant dominant-negative (DN)-PKC-β but not DN-PKC-α or DN-PKC-δ attenuated high-concentration glucose-induced tyrosine phosphorylation of VE-cad. **C**, Overexpression of DN-PKC-β but not DN-PKC-α or DN-PKC-δ reduced basal-level tyrosine phosphorylation of VE-cad. **D**, Overexpression of recombinant dominant-negative (DN)-PKC-β but not DN-PKC-α or DN-PKC-δ attenuated high-concentration glucose-induced dissociation of β-catenin from VE-cadherin complex. VE-cad antibody was used for immunoprecipitation, and β-catenin and VE-cad antibodies were used for the detection of each respective protein. mIgG (mouse non-immune IgG) was used as an immunoprecipitation control. *P < 0.05, **P < 0.01, ***P < 0.001 vs. control. Each experiment was independently performed 3 to 4 times.

Activation of PKC contributes to microvascular barrier dysfunction [19] and inhibition of PKC-β reverses cardiac microvascular barrier dysfunction in diabetic rats [20]. We recently demonstrated that the treatment of HUVECs with the PKC activator PMA increases the tyrosine phosphorylation of VE-cad, the dissociation of β-catenin from the VE-cad complex, and the TEM of invasive breast cancer cells, MDA-MB-231 cells [21]. Therefore, we hypothesized that PKC plays a role in the high-concentration glucose–induced disruption of endothelial AJs. We found that the treatment of HUVECs with high-concentrations of glucose led to a dose-dependent increase in the activation of endothelial PKC (Figure 2) and the phosphorylation of ERK (Figure 2B), which leads to MLC phosphorylation and VE-cad tyrosine phosphorylation [9]. In addition, high-concentration glucose–induced tyrosine phosphorylation of VE-cad was attenuated when HUVECs were treated with bisindolylmaleimide, an inhibitor of PKC (Figure 2C). Treatment of HUVECs with 50 nM PMA for 48 h resulted in a significant reduction in PKC activity (Figure 2D). Furthermore, when HUVECs were depleted of PKC by lengthy treatment with PMA (48 h), the high-concentration glucose–induced TEM of THP-1 cells was reduced (Figure 2E). In addition, the inhibition of intracellular calcium by BAPTA-AM (N',N'-tetraacetic acid) attenuated the high-concentration glucose-induced tyrosine phosphorylation of VE-cad (Figure 3A), suggesting a role for conventional PKC (ie, PKC-α, −β1, −β2, and -γ) in the high-concentration glucose-induced tyrosine phosphorylation of VE-cad. Similar experiments with HAECs demonstrated that PKC mediates high glucose-induced tyrosine phosphorylation of VE-cad (data not shown). When PKC-β, PKC-α, or PKC-δ was inhibited by overexpression of the corresponding dominant-negative protein, only PKC- β abolished high-concentration glucose–induced VE-cad tyrosine phosphorylation (Figure 3B). We also found that the inhibition of PKC-β but not the inhibition of PKC-α or PKC-δ by overexpression of the corresponding dominant-negative protein inhibited basal levels of VE-cad tyrosine phosphorylation by 25% (Figure 3C). Inhibition of PKC-β but not of PKC-α or PKC-δ by over expression of the corresponding dominant-negative protein resulted in suppression of high glucose induced dissociation of β-catenin from VE-cadherin complex (Figure 3D). These results suggest that high-concentration glucose-induced VE-cad tyrosine phosphorylation is mediated by PKC-β.

### Phosphorylation of MLC Mediates Tyrosine Phosphorylation of VE-cad Induced by High-Concentration Glucose

**Figure 4:**
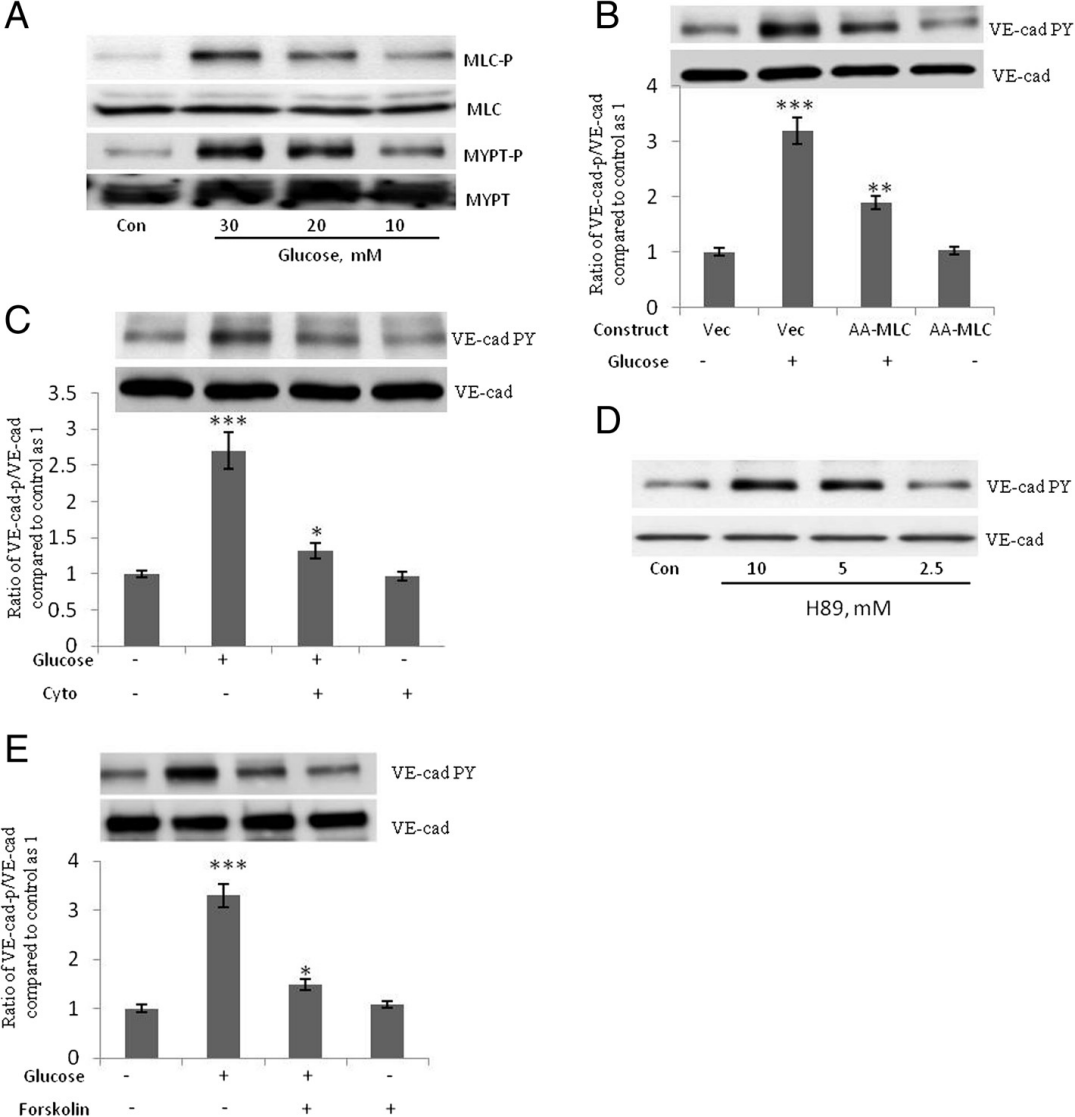
**Phosphorylation of myosin light chain (MLC) mediates high-concentration glucose-induced tyrosine phosphorylation of VE-cad. A**, Treatment of human umbilical vein endothelial cells (HUVECs) with glucose for 24 h led to phosphorylation of MLC and MLC phosphatase regulatory subunit (MYPT). **B**, High-concentration glucose-induced VE-cad tyrosine phosphorylation was attenuated in HUVECs overexpressing mutant MLC (AA-MLC), in which Thr18 and Ser19 are replaced with alanines. HUVECs were transfected with the indicated constructs and, after 48 h, were treated with 20 mM glucose for 24 h. **C**, Cytochalasin **D** suppressed high**-**concentration glucose-induced tyrosine phosphorylation of VE-cad. HUVECs were treated with 20 mM glucose for 24 h and then 1 μM cytochalasin D for 2 h. **D**, Treatment of HUVECs with protein kinase A inhibitor H89 for 2 h led to tyrosine phosphorylation of VE-cad. **E**, Treatment of HUVECs with 10 μM forskolin for 2 h (an activator of adenylate cyclase) attenuated high-concentration-induced tyrosine phosphorylation of VE-cad. HUVECs were first treated with 20 mM of glucose for 24 h. *P < 0.05, ***P < 0.001 vs. control. Each experiment was independently performed 3 to 4 times.

**Figure 5:**
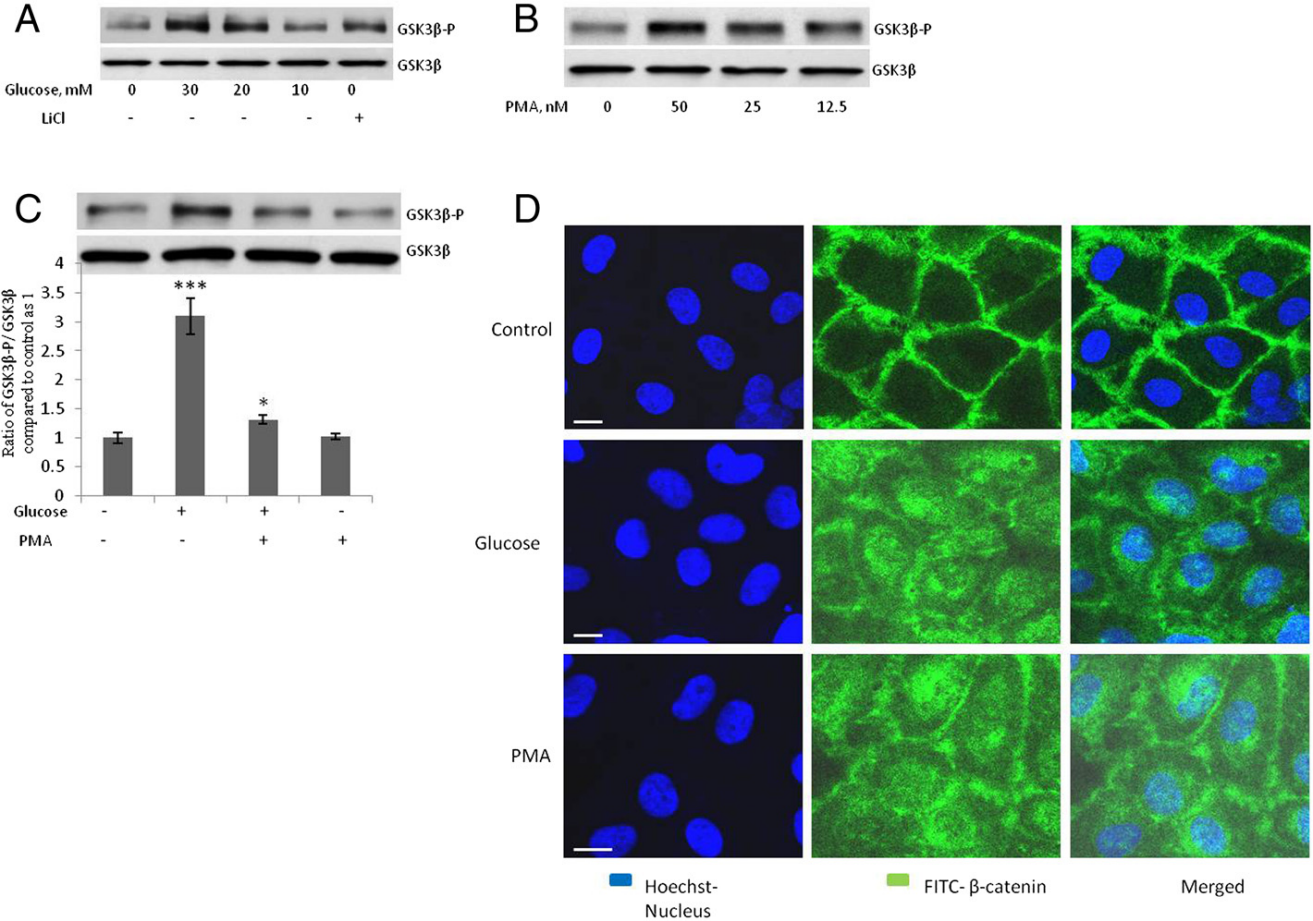
**High-concentration glucose induces the phosphorylation of GSKβ (Ser9) and the intracellular accumulation of b-catenin. A**, **B**, Treatment of human umbilical vein endothelial cells (HUVECs) with glucose (10–30 mM, 24 h), LiCl (10 mM, 4 h), or PMA (12.5-50 nM, 4 h) increased phosphorylation of GSK3β (Ser 9). **C**, Treatment of HUVECs with 50 nM of PMA for 48 h attenuated high-concentration-glucose-induced GSK3β phosphorylation. After treatment of the indicated samples with PMA for 48 h, glucose was added to the indicated samples for 24 h before GSK3β (Ser 9) phosphorylation was evaluated. **D**, Treatment of HUVECs with 20 mM of glucose for 24 h or 50 nM of PMA for 4 h induced the translocation of β-catenin into the cytoplasm and nuleus of the cells. HUVECs were fixed with 4% paraformaldehyde and stained with primary rabbit polyclonal antibody against β-catenin and secondary FITC-conjugated goat anti-rabbit IgG antibody (green). Hoechst was used to stain the nuclei (blue). Bar = 10 μm. Magnification, 63X. ***P < 0.001, **P < 0.01, and *P < 0.05 vs. control. Each experiment was independently performed 3 to 4 times.

Because MLC phosphorylation plays a crucial role in VE-cad tyrosine phosphorylation induced by the interaction of monocytes with endothelial cells [9] we studied the role of MLC phosphorylation and actin polymerization in the high-concentration glucose-induced tyrosine phosphorylation of VE-cad. The high-concentration glucose-induced tyrosine phosphorylation of VE-cad was accompanied by an increase in the phosphorylation of MLC and the regulatory subunit of MLC phosphatase (MYPT, Figure 4A). Phosphorylation of MYPT inhibits MLC phosphatase [22]. A nonphosphorylatable mutant construct for MLC in which Thr18 and Ser19 are replaced with alanines (AA-MLC) was used to study the role of MLC phosphorylation in high-concentration glucose-induced tyrosine phosphorylation of VE-cad [9,17]. The inhibition of MLC phosphorylation by the overexpression of DN-AA-MLC (GFP transfection rate, 40%; Figure 4B) and the inhibition of actin polymerization by cytochalasin D (1 μM, 2 h; Figure 4C) attenuated the high-concentration glucose-induced tyrosine phosphorylation of VE-cad, suggesting a crucial role for MLC phosphorylation in mediating the effects of high-concentration glucose on the tyrosine phosphorylation of VE-cad. Studies with HAECs indicated that induction of VE-cad tyrosine phosphorylation by high glucose is mediated by MLC phosphorylation (data not shown). Given that PKA inhibits MLC phosphorylation [23] we examined the effects of PKA inhibitor H89 on VE-cad phosphorylation. H89 increased VE-cad tyrosine phosphorylation in HUVECs in a dose-dependent manner (Figure 4D). Furthermore, because the cAMP/PKA signaling pathway stabilizes the barrier property of endothelial cells [24,25] we examined the effect of increasing the concentration of cAMP on the high-concentration glucose-induced tyrosine phosphorylation of VE-cad. In HUVECs treated with forskolin (an activator of adenylate cyclase), the high-concentration glucose-induced tyrosine phosphorylation of VE-cad was attenuated (Figure 5E). These results suggest that the high-concentration glucose-induced tyrosine phosphorylation of VE-cad is mediated by MLC phosphorylation.

### High-Concentration Glucose Induces Phosphorylation of GSK3β and Transactivation of β-catenin–Responsive Genes

Dissociation of AJs leads to the internalization of cadherin-bound β-catenin and its accumulation in the perinuclear endocytic recycling compartment [26]. Upon stimulation of the wingless (Wnt) signaling pathway, glycogen synthase kinase-3β (GSK3β), a protein that is part of the complex that regulates the proteasomal degradation of β-catenin, is inactivated/phosphorylated, and β-catenin is translocated to the nucleus where it associates with Lef-1/TCF to activate target gene transcription and biological responses [27,28].To determine if Wnt- β-catenin pathway is activated after high-concentration glucose-induced dissociation of β-catenin from VE-cad, we studied the phosphorylation of GSK3β and the cellular localization of β-catenin. The treatment of HUVECs with high concentrations of glucose (24 h) or PMA (4 h) resulted in the dose-dependent inactivation/phosphorylation of GSK3β (Figure 5A and B). GSK3β was also phosphorylated when HUVECs were treated with LiCl, a known inhibitor of GSK3β (Figure 5A). When HUVECs were depleted of PKC by lengthy treatment (48 h) with PMA, the high-concentration glucose-induced phosphorylation of GSK3β was significantly reduced (Figure 5C), suggesting that PKC mediates high-concentration glucose-induced GSK3β phosphorylation. The treatment of HUVECs with high-concentration glucose or PMA (4 h) resulted in the accumulation of intracellular β-catenin (Figure 5D).

**Figure 6:**
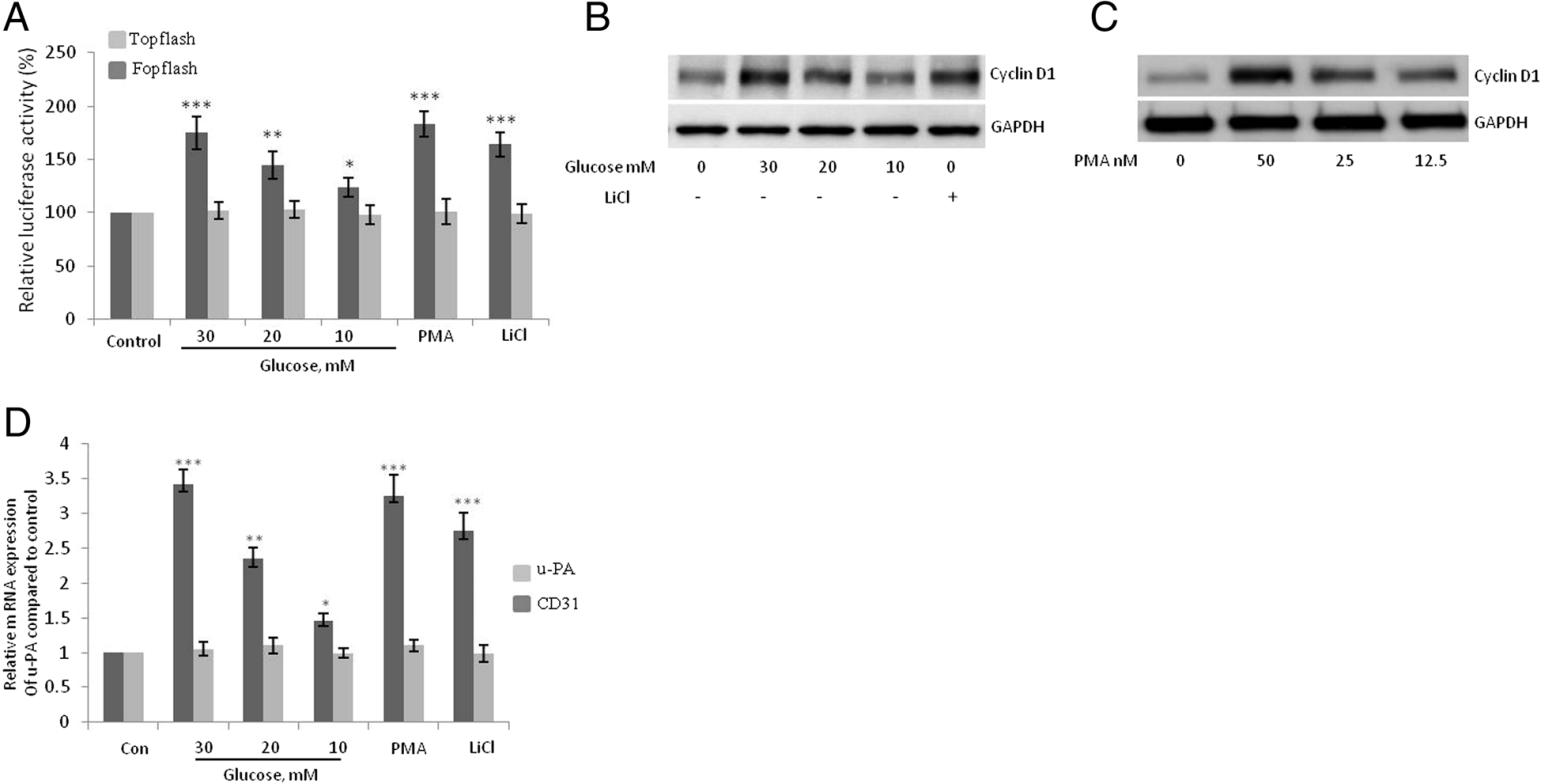
**High-concentration glucose induces the transactivation of β-catenin-responsive genes. A**, Glucose (10–30 mM, 24 h), PMA (50 nM, 8 h), and LiCl (10 mM, 8 h) induced an increase in TOPflash but not mutant FOPflash luciferase activity in human umbilical vein endothelial cells (HUVECs). Three independent experiments were performed. Luciferase activity was normalized to that of Renilla and is expressed as a percentage of the control. **B**, **C**, Western blot analysis showing that the treatment of HUVECs with glucose (24 h), LiCl (10 mM, 16 h) or PMA (16 h) increased protein expression of cyclin D1. **D**, Real-time PCR analysis showing that the treatment of HUVECs with glucose (10–30 mM, 24 h), LiCl (10 mM, 8 h), or PMA (50 nM, 8 h) increased mRNA expression of urokinase plasminogen activator, u-PA but not the endothelial cell marker CD31. Expression levels for the u-PA gene were normalized to those of the housekeeping gene β2-microglobulin. Data are expressed as the mean ± SD of 4 independent experiments. ***P < 0.001; **P < 0.01, and *P < 0.05 vs. control. Each experiment was independently performed 3 to 4 times.

To determine whether high-concentration glucose induces the transactivation of β-catenin-responsive genes, we performed luciferase reporter assays using luciferase reporters that contain either the wild-type (TOPflash) or mutated (FOPflash) binding sites for the Lef-1/TCF complex, which have been widely used to characterize β-catenin-TCF-dependent gene expression [29]. High-concentration glucose, PMA, or LiCl significantly increased TOPflash Lef-1/TCF-responsive promoter activity but had no effect on FOPflash promoter activity (Figure 6A). Furthermore, the treatment of HUVECs with high-concentration glucose, PMA, or LiCl increased the protein expression of cyclin D1, which is encoded by a β-catenin–responsive gene (Figure 6B and C). In addition, the mRNA expression of another β-catenin–responsive gene, urokinase plasminogen activator, u-PA but not the endothelial cell marker CD31, was also increased after HUVECs were treated with high-concentration glucose, PMA, or LiCl (Figure 6D). These results suggest that the Wnt/β-catenin pathway is activated in endothelial cells treated with high concentrations of glucose.

## Discussion

In our study, we showed that the treatment of endothelial cells with high-concentration glucose led to the tyrosine phosphorylation of VE-cad, the dissociation of β-catenin from the VE-cad complex, and the increased TEM of monocytes. Furthermore, the disruption of endothelial AJ integrity induced by high-concentration glucose was mediated by PKC-β-dependent MLC phosphorylation and was associated with the activation of the Wnt/β-catenin signaling pathway. Our findings provide evidence of a mechanism that may underlie the increased risk of atherosclerosis in patients with diabetes.

Recent studies in low-density lipoprotein receptor–deficient mice, which are hyperglycemic but exhibit no marked dyslipidemia when compared with nondiabetic controls, showed that diabetes in the absence of diabetes-induced hyperlipidemia is associated with the accelerated formation of atherosclerotic lesions, similar to what is seen in fat-fed nondiabetic mice [30]. The observed increase in the adhesion of leukocytes to endothelial cells has been attributed to the hyperglycemia-induced overexpression of endothelial adhesion molecules [31]. Our studies suggest that the high-concentration glucose–induced disruption of endothelial AJs may also be a mechanism underlying accelerated atherosclerosis in diabetes. Based on the results of our previous studies [9,16,17,32] we have developed a hypothetical model [9] for how the interaction of monocytes with endothelial cells leads to the enhancement of monocyte TEM. We showed that increased phosphorylation of MLC by inhibition of MLC phosphatase leads to tyrosine phosphorylation of VE-cad [9]. Several signaling pathways regulate MLC phosphorylation, including PKC, Rho-family GTPases, and RAS [32-37]. Therefore, we logically speculated that the same pathways regulate VE-cad tyrosine phosphorylation. Our previous and current findings support this hypothesis [9,16,17]. However, further investigation is needed to identify the underlying mechanisms by which high-glucose-induced phosphorylation of MLC leads to VE-cad tyrosine phosphorylation. It is possible that MLC phosphorylation and actomyosin contraction trigger not only mechanical and cytoskeletal changes in endothelial cells but also lead to the redistribution of kinases and phosphatases that regulate VE-cad tyrosine phosphorylation. Such a mechanism may contribute to the phosphorylation of VE-cad in endothelial cells treated with high-concentration glucose. Previous studies showed that actin polymerization, RhoA activity, and myosin activity are required for the recruitment and accumulation of junctional complex components [38,39]. Two nonreceptor tyrosine kinases that regulate the tyrosine phosphorylation of VE-cad-Src and Pyk2 [9] -associate with VE-cad [40,41]. We showed that the interaction of monocytes with endothelial cells or the overexpression of constitutively active HRas in endothelial cells recruits Src to the VE-cad complex [9]. In this study, we showed that the treatment of HUVECs with high-concentration glucose resulted in the phosphorylation/inhibition of GSK3β, which in turn led to the accumulation of intracellular β-catenin, an increase in TCF-responsive promoter (TOPflash) activity, and the transactivation of the β-catenin–responsive genes cyclin D1 and u-PA. It has been shown that u-PA and its cell surface receptor, u-PAR, are overexpressed in atherosclerotic plaques and may contribute to vascular wall inflammation [42]. In addition, in vivo microscopy analysis of the cremaster muscle in mice showed that endothelial u-PA promotes the TEM of neutrophils [43]. Vascular endothelial growth factor (VEGF), which is known to increase VE-cad tyrosine phosphorylation [44], induces the hyperpermeability of endothelial cells by activating the u-PA/u-PAR system through the transcriptional activation of β-catenin, thus increasing u-PAR expression [45]. Moreover, u-PA increases the permeability of endothelial cells [46]. Interestingly, u-PA activates ERK in many different cell types including endothelial cells [47,48]. Furthermore, uPA promotes cellular migration by initiating a u-PAR–dependent signaling cascade in which Ras, MEK, ERK, and MLC kinase serve as essential downstream effectors [49].

These findings suggest that u-PA may initiate the signal transduction that leads to VE-cad tyrosine phosphorylation and the disruption of AJs. Additional studies are needed to determine the role of high-concentration glucose-induced u-PA expression in endothelial AJ integrity.

## Conclusions

We showed that treatment of endothelial cells with high-concentration glucose leads to the PKC-β–mediated disruption of endothelial AJs and the activation of the Wnt/β-catenin signaling pathway. The results of this study provide supportive *in vitro* evidence for a possible molecular mechanism underlying the increased risk of atherosclerosis in diabetes.

## Competing interests

The authors declare that they have no competing interests.

## Authors’ contributions

WZ carried out cell culture, western blot and protein kinase C assays. MH conceived of the study, carried out the cellular and molecular studies, prepared the manuscript pictures, performed statistical analysis, and drafted and finalized the manuscript. JW and RD participated in the design of the study and helped to draft and edit the manuscript. All authors read and approved the final manuscript.
